# Computerised patient-specific prediction of the recovery profile of upper limb capacity within stroke services: the next step

**DOI:** 10.1136/jnnp-2020-324637

**Published:** 2021-01-21

**Authors:** Ruud W Selles, Eleni-Rosalina Andrinopoulou, Rinske H Nijland, Rick van der Vliet, Jorrit Slaman, Erwin EH van Wegen, Dimitris Rizopoulos, Gerard M Ribbers, Carel GM Meskers, Gert Kwakkel

**Affiliations:** 1 Rehabilitation Medicine & Plastic and Reconstructive Surgery, Erasmus MC - University Medical Center Rotterdam, Rotterdam, Netherlands; 2 Biostatistics, Erasmus MC - University Medical Center Rotterdam, Rotterdam, Netherlands; 3 Rehabilitation Research Centre, Reade, Amsterdam, Netherlands; 4 Rehabilitation Medicine, Erasmus MC - University Medical Center Rotterdam, Rotterdam, Netherlands; 5 Neuroscience - University Medical Center Rotterdam, Erasmus MC, Rotterdam, Netherlands; 6 Rijndam Rehabilitation Center, Rotterdam, Netherlands; 7 Rehabilitation Medicine, Amsterdam UMC - Location VUMC, Amsterdam Movement Sciences, Amsterdam, Netherlands

**Keywords:** stroke, stroke unit, upper extremity, outcome measure, prognosis, models, biostatistics, biomarkers

## Abstract

**Introduction:**

Predicting upper limb capacity recovery is important to set treatment goals, select therapies and plan discharge. We introduce a prediction model of the patient-specific profile of upper limb capacity recovery up to 6 months poststroke by incorporating all serially assessed clinical information from patients.

**Methods:**

Model input was recovery profile of 450 patients with a first-ever ischaemic hemispheric stroke measured using the Action Research Arm Test (ARAT). Subjects received at least three assessment sessions, starting within the first week until 6 months poststroke. We developed mixed-effects models that are able to deal with one or multiple measurements per subject, measured at non-fixed time points. The prediction accuracy of the different models was established by a fivefold cross-validation procedure.

**Results:**

A model with only ARAT time course, finger extension and shoulder abduction performed as good as models with more covariates. For the final model, cross-validation prediction errors at 6 months poststroke decreased as the number of measurements per subject increased, from a median error of 8.4 points on the ARAT (Q1–Q3:1.7–28.1) when one measurement early poststroke was used, to 2.3 (Q1–Q3:1–7.2) for seven measurements. An online version of the recovery model was developed that can be linked to data acquisition environments.

**Conclusion:**

Our innovative dynamic model can predict real-time, patient-specific upper limb capacity recovery profiles up to 6 months poststroke. The model can use all available serially assessed data in a flexible way, creating a prediction at any desired moment poststroke, stand-alone or linked with an electronic health record system.

## Introduction

Predicting poststroke motor recovery is important to set realistic and feasible treatment goals, select effective therapies and determine adequate discharge policy and long-term management.^1^ In addition, prediction models can assist in clinical trial design to select patients that may benefit most for the treatment of interest; for example, by including only those patients that have the potential to benefit from the intervention under study.^2 3^ For health economics, prediction models allow benchmarking or help correct for casemix variability and for estimating the expected costs in healthcare.^1^

Until today, a large number of prediction models for poststroke upper limb motor recovery have been developed (see for a review, see reference 2). Most of these models are based on clinical markers, such as voluntary control of shoulder abduction and finger extension (SAFE model) early poststroke^4^ (for a review, see reference 5). Other models include neurophysiological markers such as intactness of the corticospinal pathway, measured with, for example, transcranial magnetic stimulation (TMS) (for review, see references 6 7).

Typically, current prediction models are developed with classic linear or logistic regression techniques. These models assume that candidate determinants and recovery outcomes are measured at predefined times poststroke; for example, determinants assessed at 48 hours poststroke are used to predict recovery at 6 months poststroke.^2^ Classic regression approaches, however, have several limitations. First, the accuracy of the prediction depends on the ability to assess patients at predefined timepoints poststroke.^3^ In clinical practice, measuring at predefined timepoints is challenging for many practical reasons. A second limitation of classic regression models is that they only predict one specific follow-up moment, for example, at 3 or 6 months poststroke. To predict an outcome at any given moment poststroke would require a large number of regression models. Third, a limitation of regression-based models is that a nonlinear time course of recovery is not taken into account, as regression models are based on only two timepoints (ie, baseline and outcome).

Since prospective cohorts of stroke recovery with serial measurements have shown both non-linearity and high interindividual variability of stroke recovery,^4 8^ as an alternative to regression models, mixed models can be used. Mixed models are ideally suited when the individual trajectory of recovery over time is influenced by characteristics that vary from patient to patient and explicitly account for the correlations between repeated measurements within each individual patient.^5^ In a mixed model, data from all assessments contribute to more precise estimates and characterise an individual patient’s recovery pattern over time.^9^ Thus, with mixed models, more precise predictions may be achieved by including multiple repeated measurements from an individual patient, allowing patient-specific predictions with limits of uncertainty and taking all serially assessed information of that subject into account. In such a model, each new data point with new health status information will decrease the uncertainty of the individual prediction, taking into account changes in neurological status of the patient (‘dynamic prediction’).

In the current study, we aimed to develop an online-available mixed-effects prediction model for recovery in the first 6 months poststroke of the upper limb capacity, the ability to performunilateral tasks like picking up a cup, transporting blocks, manipulating small objects, measured with the Action Research Arm Test (ARAT) scores. Subsequently, we investigated how this dynamic model can take time-dependent clinical improvement of patients into account as soon as new information is available. After comparing several model alternatives, we cross-validated the accuracy of the predictions and developed an online patient-specific model in which the predicted outcome can be used at stroke units and throughout the care chain for predicting the recovery profile of upper limb capacity.

## Materials and methods

### Patients and measurements

We combined data on upper extremity capacity recovery in first-ever ischaemic anterior circulation stroke patients from four early poststroke prospective cohort studies with repeated measurements in time: the EXPLICIT,^3^ EPOS,^10^ 4D-EEG^11^ and EXPLORE cohorts.

All datasets contain repeated measurements of the ARAT scores after the stroke. Patients in the 4D-EEG and EXPLORE cohorts were recruited within 3 weeks poststroke. Patients in the 4D-EEG study and the EXPLORE study were measured in the first week poststroke and after 5, 12 and 26 weeks. In the EXPLICIT study, patients were measured 1, 2, 3, 5, 12 and 26 weeks poststroke and in the EPOS study in the first 3 days, at day 5 and 9 and after 6 months. All studies included adult patients with a monoparesis or hemiparesis within the first 72 hours after stroke onset, without disabling medical history and with no severe deficits in communication, memory, or understanding that impede proper measurement performance.

Patients received standard rehabilitation treatment according to the Dutch rehabilitation guidelines, which are in agreement with current international rehabilitation guidelines.^12 13^ In the EXPLICT study (N=159), half of the patients with an unfavourable prognosis received electromyography-triggered neuromuscular stimulation and half of the patients with a favourable prognosis received modified constrain-induced movement therapy.^3^

### Testing covariates

In line with recent international recommendations,^14^ upper extremity capacity measured with the ARAT was used as the dependent variable in the model. The ARAT has 19 tasks divided into four subdomains: grasp, grip, pinch and gross movement. The ARAT score ranges from 0 to a maximal score of 57 points, and the ARAT has excellent clinimetric properties.^15 16^

We included the following potential demographic and clinical covariates variables during the model development: age, gender, affected bodyside, dominant side, Bamford scale (lacunar cerebral infarct/partial anterior circulation infarcts/total anterior circulation infarcts),^17^ and administration of alteplase (rt-PA). Neurological deficits were assessed with the National Institutes of Health Stroke Scale (NIHSS; range 0–42). NIHSS item 8 reflecting somatosensory deficit (range 0–2) and item 11 measuring extinction and inattention (range 0–2) were used as separate covariates.^18^ In addition, we used the O-letter cancellation test^19 20^ to assess visuospatial neglect using a cut-off of 2 missing O’s or more on the hemiplegic side.

As time-dependent variables, we included the Fugl Meyer Upper Extremity (FM-UE) motor score (range 0–66).^17^ In addition, the item voluntary finger extension (FE: no=0 or yes=1 or 2 points) of the FM-UE was used as a separate item for deriving the SAFE model. Upper and lower limb strength were measured with the Motricity Index leg and the Motricity Index arm score (range 0–99).^21 22^ The item ‘shoulder abduction’ was also used separately (six levels, range 0–33) for the SAFE model.

### Prediction model development

For model fitting, we included all patients with at least three repeated measurements from which the first and last measurements were at least 12 weeks apart. We excluded patients for the model fitting who showed an FM-EU decrease beyond the upper limit of the clinically important difference for the FM-UE (7.25) in the first 6 months.^23^ We did, however, include these patients in the cross-validation to present a fair estimate of prediction accuracy.

As a first step in the model fitting, we compared mixed-effects models with different time structures to investigate whether a nonlinear time, compared with a linear time structure, was more appropriate to capture the mean recovery profile of the ARAT (fixed effects) and the subject-specific ARAT evolutions (random effects). For the models with nonlinear time structures, we used natural cubic splines with a different number of knots. The knots were placed at the quartile points in the case of 1, 2 and 3 knots and placed manually in the case of 6 knots.

Subsequently, we developed models with different fixed effect structures, starting from the most complex model with all covariates mentioned above (see also table 1). More specifically: model 1. ARAT scores as a function of all available covariates with their main effects and interaction with time; model 2. ARAT scores as a function of all covariates with all their main effects but only the significant interactions with time; model 3. ARAT scores as a function of only the significant main effects and the significant interactions with time; model 4. ARAT scores as a function of time, the SAFE model and their interaction with time and model 5. ARAT scores as a function only of time.

**Table 1:** Summary of the five different model structures that were considered

	Time dependency of ARAT alone	Covariate: all main effects	Covariate: all interaction effects (with time)	Covariate: only significant main effects	Covariate: only significant interaction effects (with time)	Covariate: only shoulder abduction and finger extension and interaction (with time)
Model 1 (most extensive model)	X	X	X			
Model 2 (all covariates, all main effects, only the significant interactions with time)	X	X			X	
Model 3 (only significant main effects and significant interactions with time)	X			X	X	
Model 4 (SAFE model)	X					X
Model 5 (minimal model)	X					

For further description of the models, see prediction model development in the methods. The safe model was used as the final model for cross-validation.

ARAT, Action Research Arm Test; SAFE, Shoulder-Abduction-Finger Extension model.

### Cross-validation and model comparison

We selected the optimal model from the above-mentioned models by comparing the prediction accuracy of the models. To express model accuracy, we calculated the absolute error between the predicted and the observed (true) ARAT measurement and presented the distribution of the errors using box-plots with the median, the IQR, the lower whisker presented as Q1 - 1.5 * IQR and the upper whisker presented as Q3 +1.5 * IQR. Values outside the range (Q1 - 1.5 * IQR, Q3 +1.5 * IQR] were considered outliers and were removed.

We performed a fivefold cross-validation procedure by splitting the data into five sets, fitted the models using four of them, and then predicting the recovery profiles for the patients from the fifth set. Although the model predicts the complete recovery profile and not only one future timepoint, to express prediction error, we compared predicted and actual recovery at 6 months poststroke. The predictions were obtained several times by adding an extra measurement for ARAT each time. We repeated this procedure 10 times.

For all analyses, R (V.3.6.1) software was used (freely available at https://cran.r-project.org). The packages nlme (V.3.1.144) and JMbayes (V.0.8.85) were used. In particular, we used the function lme() from the package nlme to fit the models and the function IndvPred_lme() from the package JMbayes to obtain the dynamic predictions.

### Online patient-specific prediction visualisation

To allow clinicians and researchers to use the prediction model for individual stroke patients, we developed an online visualisation for the real-time prediction of the recovery profile of upper extremity development and the 68% and 95% prediction intervals, expressing the prediction uncertainty. For this, we use the shiny package in R to allow uploading a text file with recorded ARAT data and covariate data, which will automatically create a patient-specific prediction of the ARAT development over time.

Within our centre, we developed an infrastructure to couple this visualisation to data capture software (GemsTracker), so that recorded data from individual patients can be used to display the predicted outcome without further data entry or analysis. Finally, we published the source code of the prediction models web-based, allowing other centres to develop similar real-time prediction tools (see details statistics and code in online supplemental file 1).

10.1136/jnnp-2020-324637.supp1 Supplementary data

## Results

### Patients and measurements

The prospective data set consisted of 450 patients with a first-ever ischaemic hemispheric stroke (see table 2), of which 52% were males, mean age at baseline was 65 years, and mean follow-up days was 166 days. Patients were serially assessed with a median of six times (25% quantile: 4; 75% quantile: 8. The mean ARAT early poststroke was 14 (SD 19); the ARAT distribution was skewed by a large sample of patients with an ARAT of 0 or 1 scores early poststroke and a less equal distribution of ARAT scores throughout the rest of the range of scores (Q1=0, median is 1, Q3 is 29, maximum score 56).

**Table 2:** Patientcharacteristics assessed within 72 hours after stroke

	Mean (SD) or %
No of patients	450
Age (years)	64.8 (14.0)
Sex (males)	52
Type of stroke (Bamford classification)	
Lacunar cerebral infarcts	48
Total anterior circulation infarcts	18
Partial anterior circulation infarcts	34
Affected body side (right)	39
Dominant hand (right)	92
Recombinant tissue plasminogen activator (yes)	23
National Institutes of Health Stroke Scale	8 (5)
Baseline Action Research Arm Test (ARAT)	14 (19)
Baseline Shoulder Abduction	
No random movement	33
Random activity palpable, no movement visible	10
Random movement seeable but not seeable in total movement range	22
Random movement across total movement range, not possible against resistance	5
Random movement against resistance, but weaker than healthy side Normal strength in comparison with contralateral side	21
Normal strength in comparison withcontralateral side	9
Fugl Meyer Upper Limb score (0–66)	25 (22)
Fugl Meyer finger extension (none, partial, full)	54, 20, 26
Motricity Index Arm (0–100)	38 (34)
Motricity Index Leg (0–100)	49 (33)
Neglect:	
Normal (not present)	63
Inattention or extinction for 1 kind of stimulus severe hemi-inattention for both stimuli	17
Severehemi-inattention for both stimuli	21
Sensibility	
Normal	45
Reduced	42
Absent	14
Baseline finger extension	
None	54
Partial	20
Full	26

**Figure 1:**
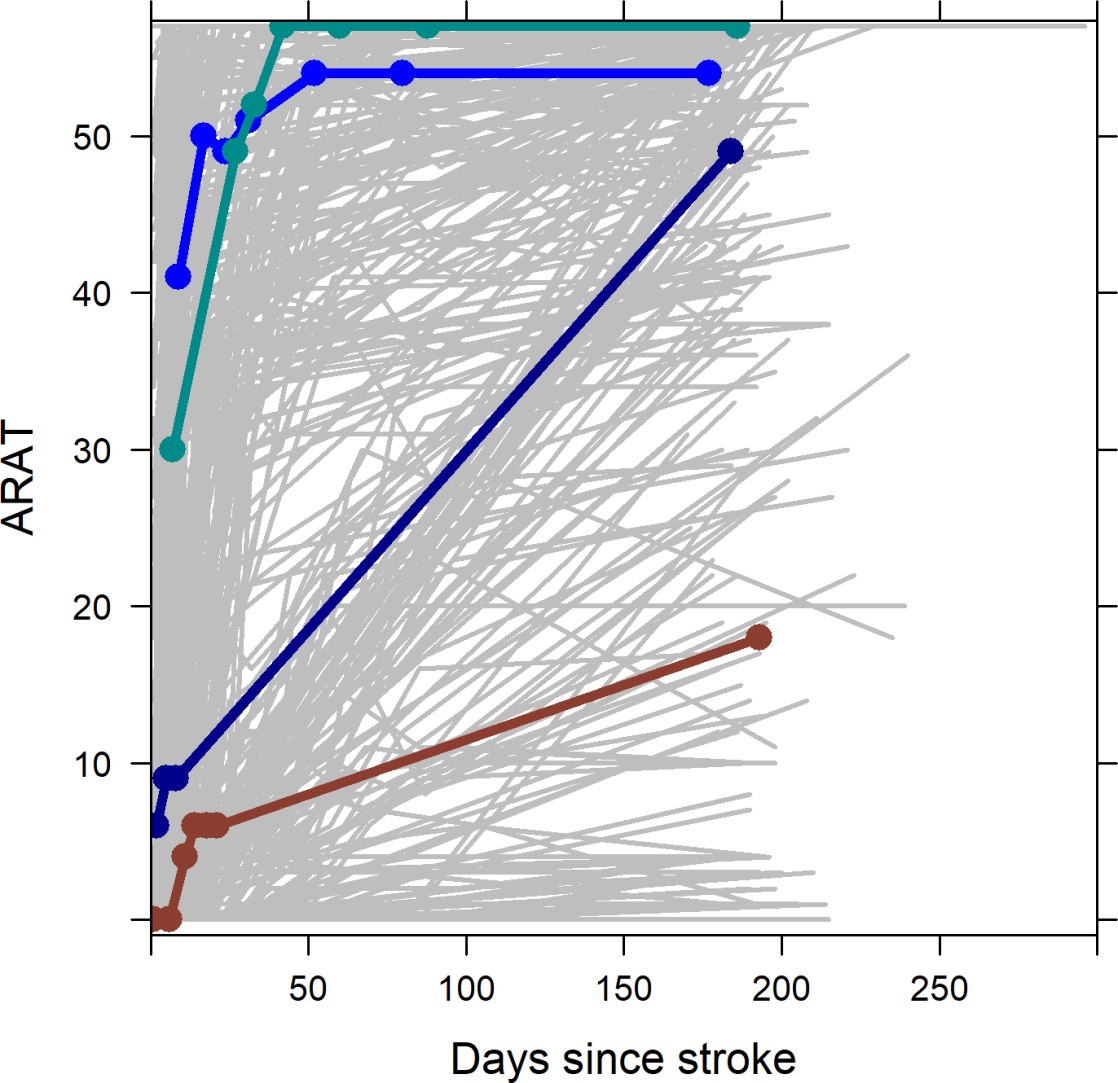
ARAT recovery profiles of all 450 patients (in grey) and four typical examples in bold and the dots indicating the exact measurement points. It can be seen that the recovery of upper extremity capacity is extremely diverse, both in terms of onset ARAT score and the change over time. Most measurements were taken approximately in the first 30 days; as a result, the change after this time point can be modelled less precisely. ARAT, Action Research Arm Test.

Figure 1 shows all 6-month recovery profiles and highlights five individual patients with different recovery profiles. The figure illustrates both the heterogeneity and non-linearity of the recovery, with some patients showing little or no recovery, others reaching the maximum ARAT score within the first 3 weeks poststroke and again others starting with a relatively high ARAT score already early after stroke and typically reaching the maximum ARAT score within a few weeks.

### Prediction model development

Visual inspection of the individual repeated measurement profiles of the ARAT (see figure 1) indicated that the final model needs to allow for nonlinear evolutions over time. After comparing models with different nonlinear evolutions, we observed small differences between using two, three and six knots (See online supplementary figure 1). However, since the main goal was to use the model for predicting the recovery profile of the ARAT most precisely, we continued with the most flexible model based on six knots. This model also provided better predictions when checking several individual prediction plots.

### Cross-validation prediction accuracy and model selection

Comparing the prediction accuracy of the different mixed-effects models and taking the first measurement of each patient as a predictor, we found mean cross-validation errors at 6 months poststroke of 10.1–105 ARAT points for the first, second and third model, 8.4 for the fourth SAFE model with only FE and SA and 17.3 for the fifth model that includes only time as a covariate (see Supplementary Figure 2 for more details). Based on these results, we decided to further continue with the fourth (SAFE) model for predicting the recovery profile of the ARAT.

**Figure 2:**
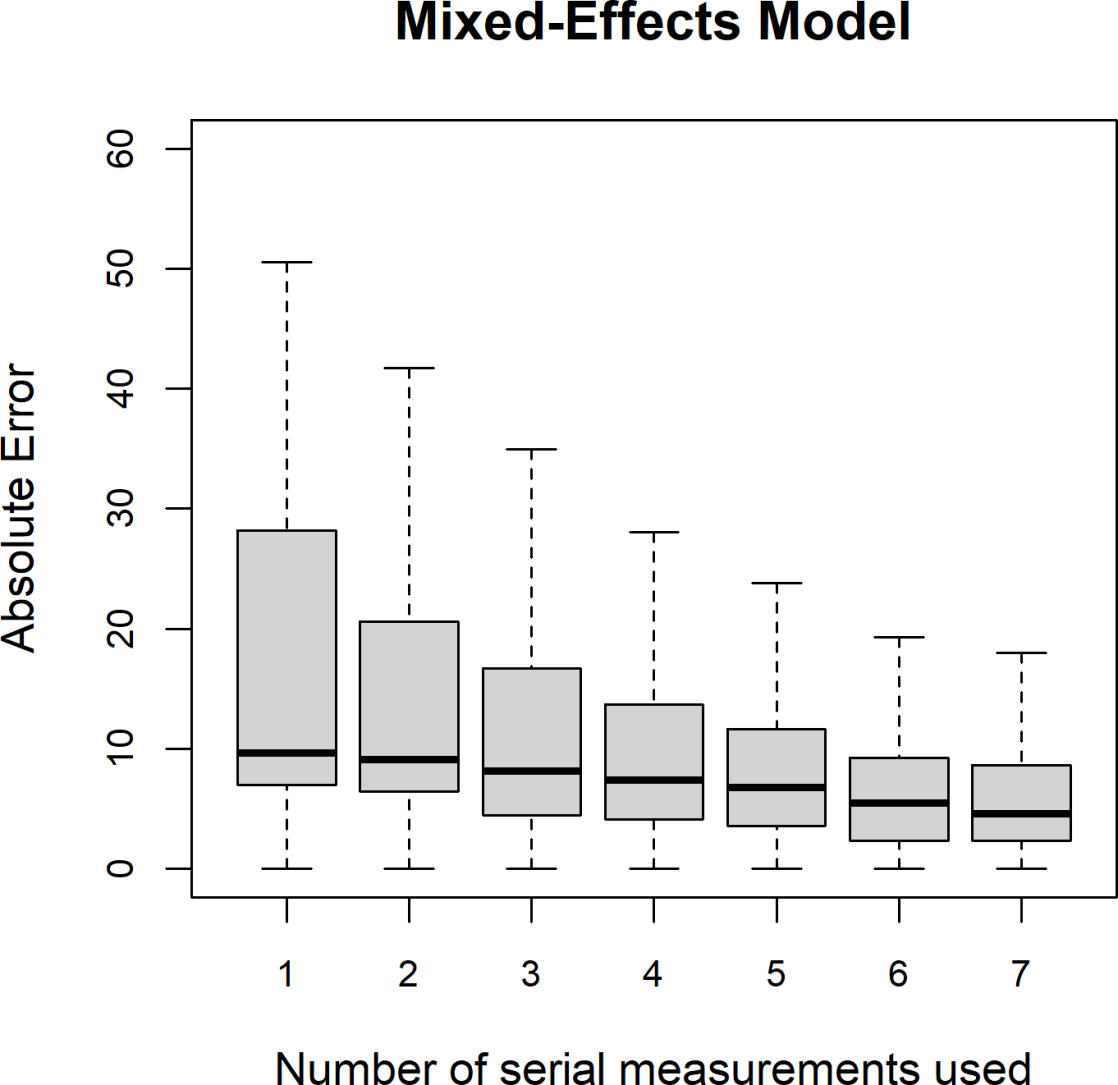
Cross-validation time-dependent accuracy of the shoulder abduction and finger extension (SAFE) model for predicting 6 months ARAT score. The accuracy was defined as the absolute difference between the predicted ARAT score at 6 months poststroke from the cross-validation and the measured ARAT score at the same time and displayed as median, (IQR: Q1=25th percentile and Q3=75th percentile), lower whisker presented as Q1–1.5 * IQR and upper whisker presented as Q3 +1.5 * IQR. The accuracy is displayed as a function of the number of serial measurements were used for predicting the outcome at 6 months, of which generally the last measurement was performed at 6 months poststroke, one at 3 months poststroke and the others between 2 days and 6 weeks poststroke. ARAT, Action Research Arm Test.

Figure 2 illustrates the prediction errors at 6 months poststroke using the SAFE model. As shown, increasing the number of serial measurements decreased the absolute error of prediction of ARAT from 8.4 points on the ARAT (Q1–Q3: 1.7–28.1) to 2.3 (Q1–Q3: 1–7.2) when seven serial measurements were used for predicting the outcome at 6 months, of which generally one measurement as 6 months poststroke, one at 3 months poststroke, and the others between 2 days and 6 weeks poststroke.

**Figure 3:**
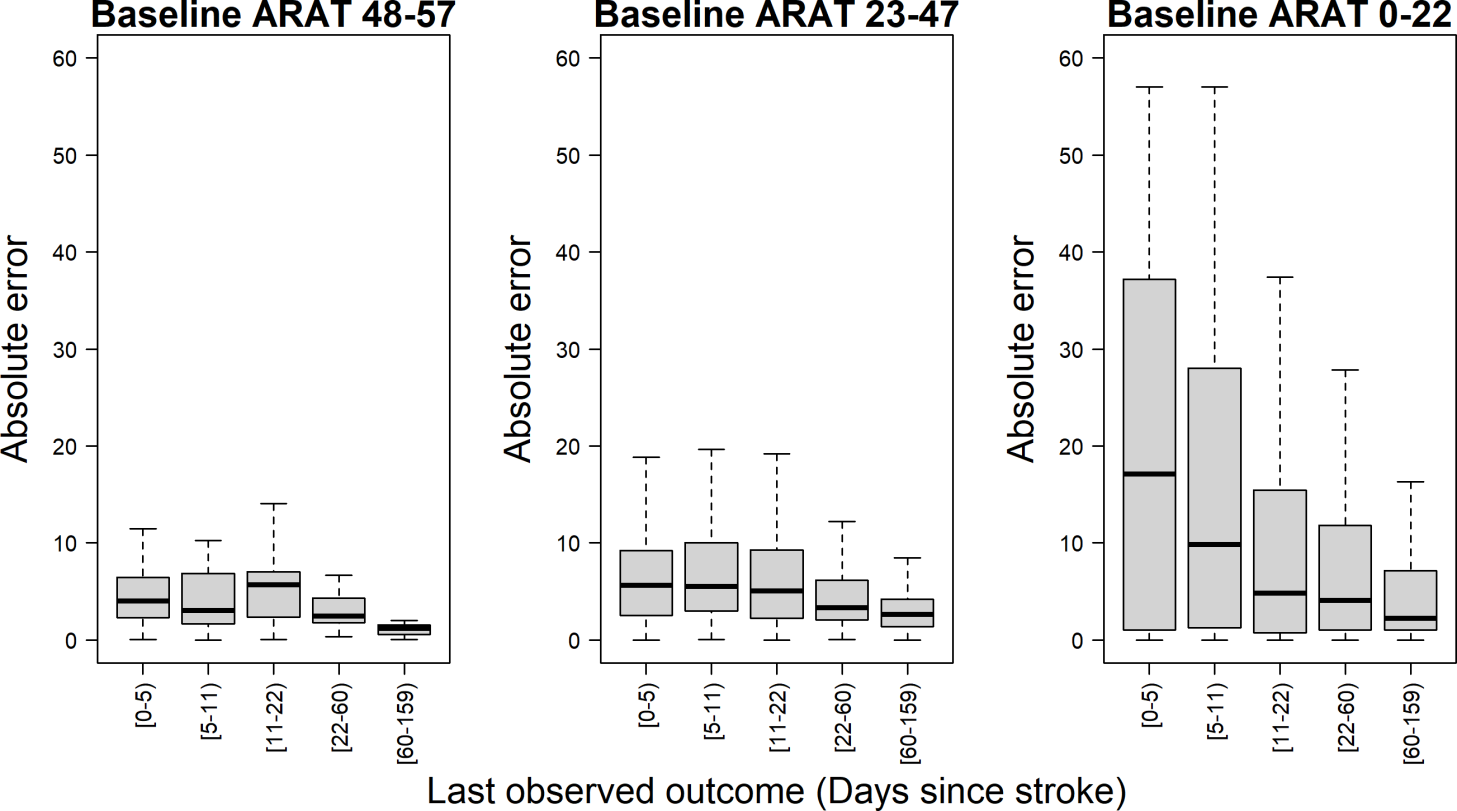
Cross-vlidation accuracy of the fivefold cross-validation in for different levels of baseline (early poststroke) ARAT scores (left, middle and right panel) for the safe model as a function of the time of the last observed outcome (instead of the number of measurements used). Furthermore, we categorised the results by baseline ARAT group. The accuracy was defined as the difference between the predicted ARAT score at 6 months poststroke from the cross-validation and the measured ARAT score at the same time and displayed as median, (IQR: Q1=25th percentile and Q3=75th percentile), lower whisker presented as Q1–1.5 * IQR and upper whisker presented as Q3 +1.5 * IQR. The results can be different from figure 3 since some patients might have one measurement during the first week while other patients might have 2–3 repeated measurements in the first 6 months. ARAT, Action Research Arm Test.

Figure 3 illustrates the prediction errors for subgroups of patients with a lower, medium and higher baseline ARAT score at different time intervals of the last observed outcome (instead of the number of measurements visualised in figure 2. The 6-month prediction error was relatively small at each time point for patients with a higher initial ARAT score, while for the patients with a low initial ARAT score, the errors were large at baseline but decreased strongly later poststroke.

### Online patient-specific prediction visualisation

**Figure 4:**
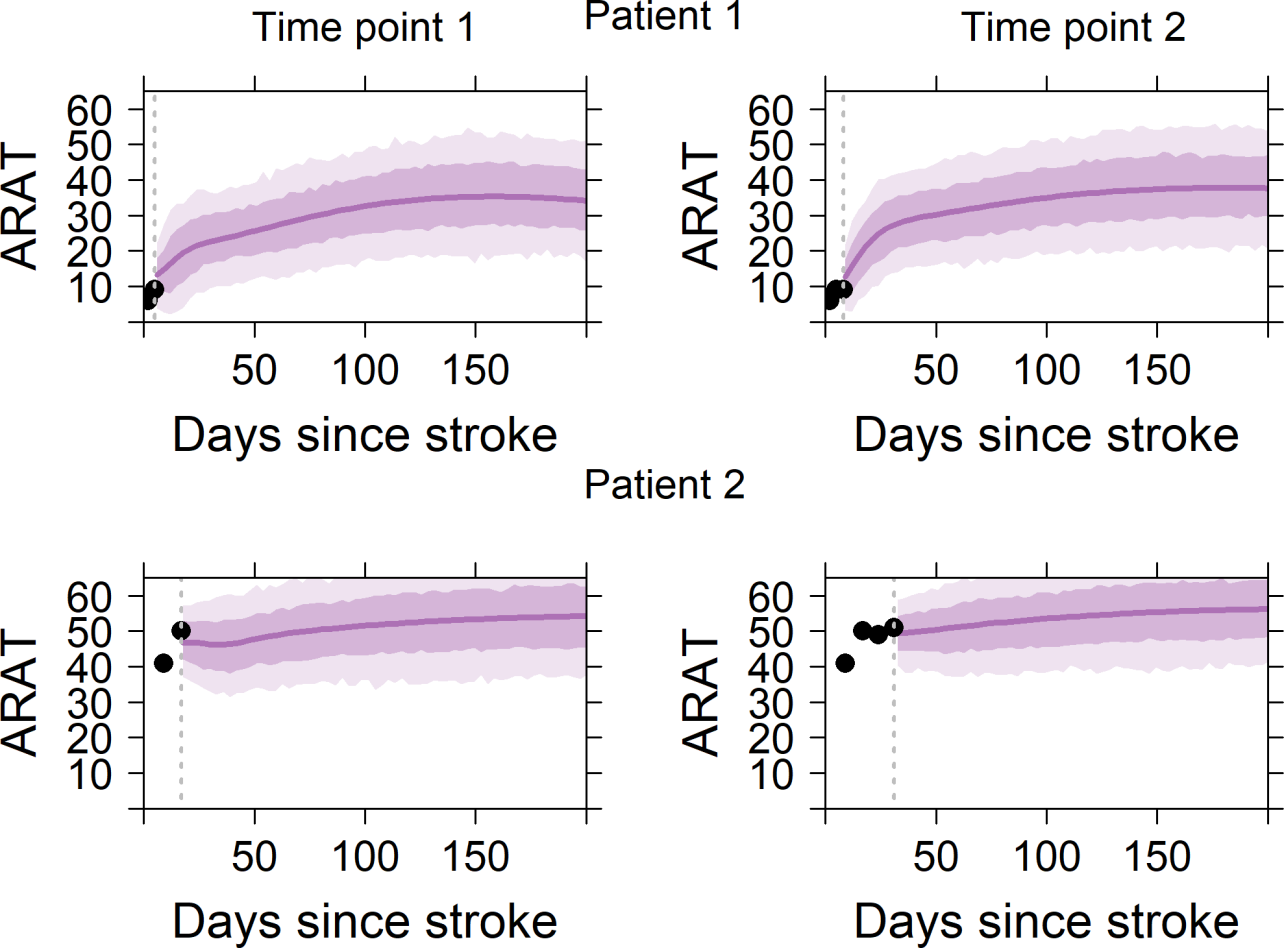
Typical examples of the predicted ARAT recovery for two patients. The dotted vertical line represents the time of the last follow-up. The circles represent all the ARAT measurements available for that patient until that specific moment, while the solid line represents the predicted ARAT recovery. The shaded areas indicate the 68% (lighter shade) and 95% (darker shade) prediction intervals. from a clinical perspective, the errors in the cross-validation provide the best estimate of what the error in predicting the outcome for a new patient will be and may, therefore, be most clinically relevant. For each patient, the predicted recovery is illustrated at a first and a second time point, not necessarily corresponding to the first and second available measurements from a patient. The data of the same patients can be downloaded in the online APP to visualised predictions at all time points: https://emcbiostatistics.shinyapps.io/DynamicPredictionARATapp https://emcbiostatistics.shinyapps.io/DynamicPredictionARATapp/. ARAT, Action Research Arm Test.

Figure 4 visualises predicted recovery profiles (with a 68% and a 95% prediction interval) using the SAFE model for two patients. The predicted recovery is nonlinear, especially in the patient with low initial ARAT scores. Patient 1 has a low ARAT scores at baseline and a moderate predicted ARAT score at 6 months. Patient 2 has a higher ARAT score at baseline and the predicted score at 6 months is >50 points. Prediction plots of the same patients as well as two additional patients, including a 68% and a 95% prediction interval, are shown in online Supplementary Figure 3.

Online access to a real-time tool to predicting upper extremity recovery for an individual patient can be found at https://emcbiostatistics.shinyapps.io/DynamicPredictionARATapp/, including instructions on how to enter data from individual patients. This is also the model that, within our own setting, is coupled to an online structured data entry system called Profits), as a model of how more computationally complex prediction models can be coupled to electronic health record data or other types of online healthcare data collection systems.

## Discussion

In the current study, we introduce an innovative mixed-effects model able to predict the patient-specific time course of upper limb recovery, including the associated limits of uncertainty within the first 6 months poststroke. We found that a model with only ARAT time course, FE and SA performed as good as models with more covariates for predicting the ARAT over time. In this final model, cross-validation prediction errors at 6 months poststroke decreased as the number of measurements per subject increased, from a median error of 8.4 points on the ARAT when one measurement early poststroke was used, to 2.3 for seven measurements. Linking this model with an electronic health record system at stroke units and rehabilitation wards will allow clinicians to obtain and visualise real time a patient’s recovery profile early poststroke.

In contrast to prognosis based on regression models, a major strength of current dynamic prediction modelling is that individual patient-specific neurological and functional recovery profiles are taken into account. By making the model accessible online and potentially connected to electronic health record systems, professionals can obtain predictions without needing to perform complex computations. At any given moment in time, such as in individual patient consultation or multidisciplinary team meetings, an individual prediction of the recovery profile of the ARAT may be generated, taking into account all available data points.

We found that a model with only time-depended improvements in SA and FE predicted recovery as accurately as more complex models that included covariates such as age, gender, the Bamford classification, the NIHSS or the full FM-UE score, which is in line with the SAFE model.^10 24 25^ In addition, we found that the predictions become more accurate when repeated clinical measurements became available in the first 8 weeks poststroke, which is in line with a recent model on FM-UE function^8^ and in line with the recent recommendations of the stroke recovery and rehabilitation round (SRRR) the Table group for clinical assessment.^14 26^ Finally, we showed that this dynamic model can be visualised and used online for clinicians working at stroke units and rehabilitation wards in an anonymised way by a simple text file as input, as shown in figures 3 and 4 and in the online application at https://emcbiostatistics.shinyapps.io/DynamicPredictionARATapp/

Since the implementation of prediction models in clinical care depends on the ease of use, an important benefit of the present algorithm is that it can be an integral part of existing electronic health record systems at stroke units, allowing direct access to the model input variables. Since the current study also shows that serial measurements in time after discharge from stroke units have an important added value in the accuracy of individual predictions, preferably, patient-specific prediction modelling should be part of electronic health record systems that span the entire chain of care of stroke services. The current prognostic study further emphasises the need for consensus on using clinically validated assessments including neuroimaging within stroke services.^27 28^ The model and visualisation approach that we propose in the study can be adapted to predict also other outcome domains using the same mixed model approach. Since integrating prediction models in many clinical settings may not be readily available and a longitudinal data set of serially measured patients may be lacking, the stand-alone online tool presented in the current study allows all clinicians easy access to this model for their own patients.

A strength of our innovative statistical approach is that it is possible to predict a patient-specific recovery profile of the upper paretic limb using only simple demographic and relatively easy to measure clinical information from electronic health record systems at stroke units. After cross-validation, we showed that the accuracy of prediction improved by retesting patients serially in time. This finding further emphasises the need for building regional stroke services in which clinicians working in the chain of settings use the same core set of clinical outcomes poststroke as recently recommended in an international consensus meeting.^29^ In the present study, we focused on predicting the ARAT as a showcase for dynamic prediction modelling. The next step will be to build patient-specific models for predicting the time course of basic ADLs and walking ability, including community walking poststroke. These steps should be seen as essential for improving current discharge policy and stroke management within current stroke services.^28^ For this purpose, preferably, the data entry should be part of the existing stroke guidelines and executed by clinicians who are trained to apply the clinical measurements in a valid way.^30^

The current study predicts the 6-month outcome on the ARAT scale based on a population of first-ever, ischaemic hemispheric strokes measured from 2005 onwards in the Netherlands. The predicted time course shown is irrespective of differences in type and intensity of rehabilitation therapies applied, which were applied according to the Dutch Stroke guidelines.^30^ Currently, the recommended amount of usual exercise therapy is only twice times 20 min per working day, which is probably insufficient to cause meaningful clinical differences in trajectories.^31^ However, one may assume that higher doses of task-specific exercise therapy may have caused beneficial effects and could serve in future models as one of the covariates.^29 31^ It is important to note, that mixed models explicitly account for the correlations between repeated measurements within each patient,^9^ avoiding the mathematical coupling found in studies that use a baseline measure to predict a change score.^32–34^ However, floor and ceiling effects, for which highly variables findings have been reported in literature (for review, see reference 35), may partly explain why prediction errors are smaller for patients with a very high or very low ARAT scores at 6 months. The high population of low ARAT scores early poststroke may decrease the quality of model fitting for patients with a moderate of high earlier ARAT scores; however, as seen in figure 3, model predictions for these patients were better than in patients with a low early ARAT score.

A limitation of our study is that we were restricted to clinical time-dependent variables alone, such as synergism, strength, voluntary shoulder and FE. With that, our clinical model has room for improvement, especially in those patients with low baseline motor scores who may or may not show neurological recovery. This is in line with previous studies indicating less accurate outcome prediction in patients with low FM-UE scores (<18 points) within the first week poststroke.^8^ We think that our current model may serve as a starting point for a more detailed stratification in recovery and rehabilitation trials, based on the patient’s potential for neurological and functional recovery poststroke. We may further refine the identified subgroups of recovery and increase precision at an individual level by targeting big data, based on the recently recommended core set of clinical outcomes recently suggested by the SRRR the Table group^29^ and in Europe.^36^ In addition, taking other time-dependent clinical covariates into account, such as somatosensory deficits and visuo-spatial neglect,^37^ the precision of individual time courses can be further improved in particular for those with a low baseline score on FM-UE. In addition, future studies may also investigate the added value of early measured neurophysiological markers such as TMS,^38 39^ Diffusion Tensor Imaging (DTI),^40 41^ structural brain scans^42^ and normalisation of directional symmetry^40^ as well as intactness of somatosensory systems in EEG^43^ for improving the precision of individual time courses for the ARAT. Another limitation is that we only have time courses up to 6-month poststroke. While this is often considered a final outcome whereafter no changes occur, several studies indicate that the ARAT score of these chronic stroke patients can be further improved with high doses of treatment.^31 44 45^ Such high dose treatment was not provided to our population and with such treatments or other successful interventions, predicted outcome can deviate from the outcome prediction provided by the current model.

A strength of our model is that we predict the exact ARAT score as well as prediction uncertainty. For clinical purposes, this level of detail may not always be needed and classifications of the ARAT scores in four different levels have been proposed^46^; such classifications could be directly calculated from our outcome prediction. Future impact studies may indicate which level of detail is needed for clinical care and how knowledge of predicted outcome for individual influences impacts the decision making in stroke rehabilitation.

## Data Availability

Data are available on reasonable request.
