# Supplementary material for: Computerised patient-specific prediction of the recovery profile of upper limb capacity within stroke services: the next step
Source: J Neurol Neurosurg Psychiatry. 2021 Jan 21;92(6):574–81. doi: 10.1136/jnnp-2020-324637 (PMC8142441; doi:10.1136/jnnp-2020-324637)
Supplement: Supplementary data [file jnnp-2020-324637supp001.pdf]

SUPPLEMENTAL MATERIAL

Computerized patient-specific prediction of the recovery profile of upper limb capacity within stroke services: The next step

Supplementary material

Statistical analysis additional information:

Mixed-effects models account for the serial evaluations of clinical parameters over time. These models can work with unbalanced datasets (i.e., datasets with an unequal number of follow-up measurements between subjects and varying times between repeated measurements of each subject), and they explicitly take into account that measurements from the same patient are more correlated than measurements from different patients.

One important aspect to investigate in mixed-effects model, is the shapes of the patient-specific evolutions over time. It is, therefore, important to postulate a mixed-effects model that appropriately captures nonlinear evolutions.

In order to assume nonlinear evolutions for the ARAT outcome, we used natural cubic splines. Splines are piecewise polynomials where we split the stroke recovery up into intervals (defined by the knots). The knots define the positions at which the piecewise polynomials meet within the recovery profile. A larger number of knots allows for a more flexible model, leading to more flexible predictions. In our application, the knots were placed at the quartile points in the case of 1, 2, and 3 knots and placed manually in the case of 6 knots.

Supplementary Figure 1: Predicted and observed values for 12 randomly-selected patients. The circles represent the observed values, while the lines the predicted values assuming different splines structure. The fitting was better when more than one knot was used, but there are small differences observed between 2, 3, and 6 knots. However, since our main goal was to predict outcome most accurately, we continued with the more flexible model with 6 knots.

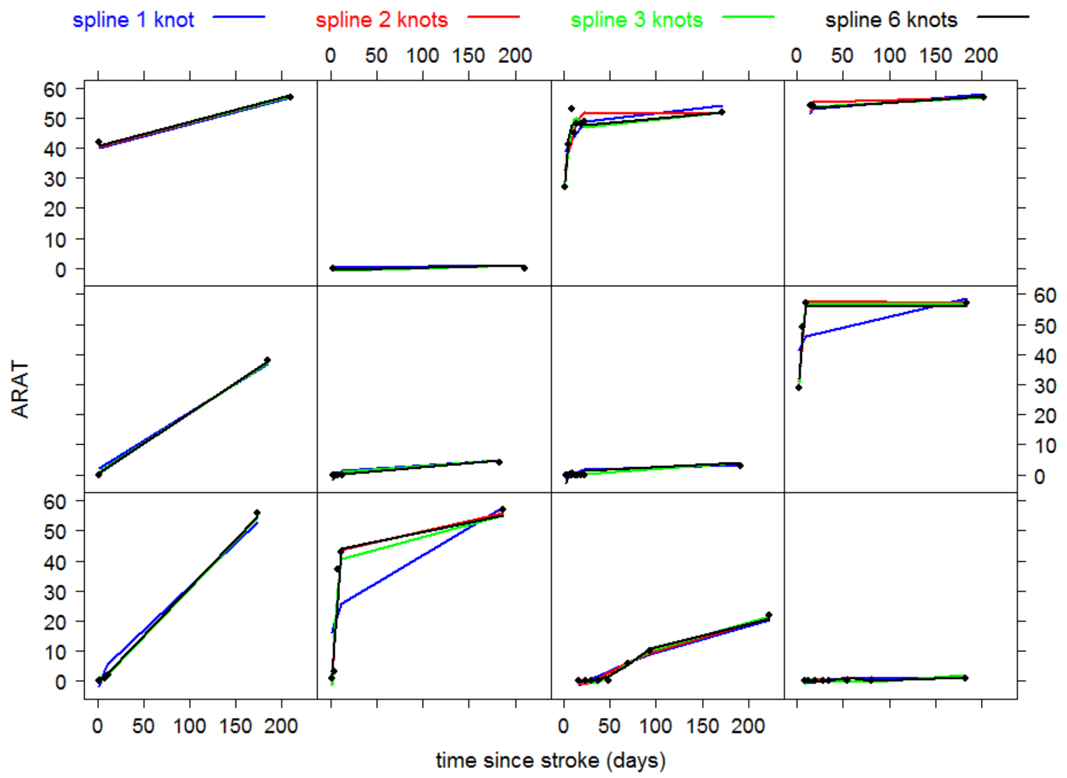

Supplementary Figure 2: Cross-validation errors of the 5 different prediction models (see *Prediction Model Development section)* as a function of the number of measurements that are used. The accuracy was defined as the absolute difference between the predicted ARAT score at 6 months poststroke from the cross-validation and the measured ARAT score at the same time and displayed as median, interquartile range (IQR: Q1 = 25th percentile and Q2 = 75 percentile), lower whisker presented as Q1 - 1.5 * IQR and upper whisker presented as Q2 + 1.5 * IQR. As can be seen, the cross-validation errors for models 1-4 are very similar and only model 5 is clearly less accurate.

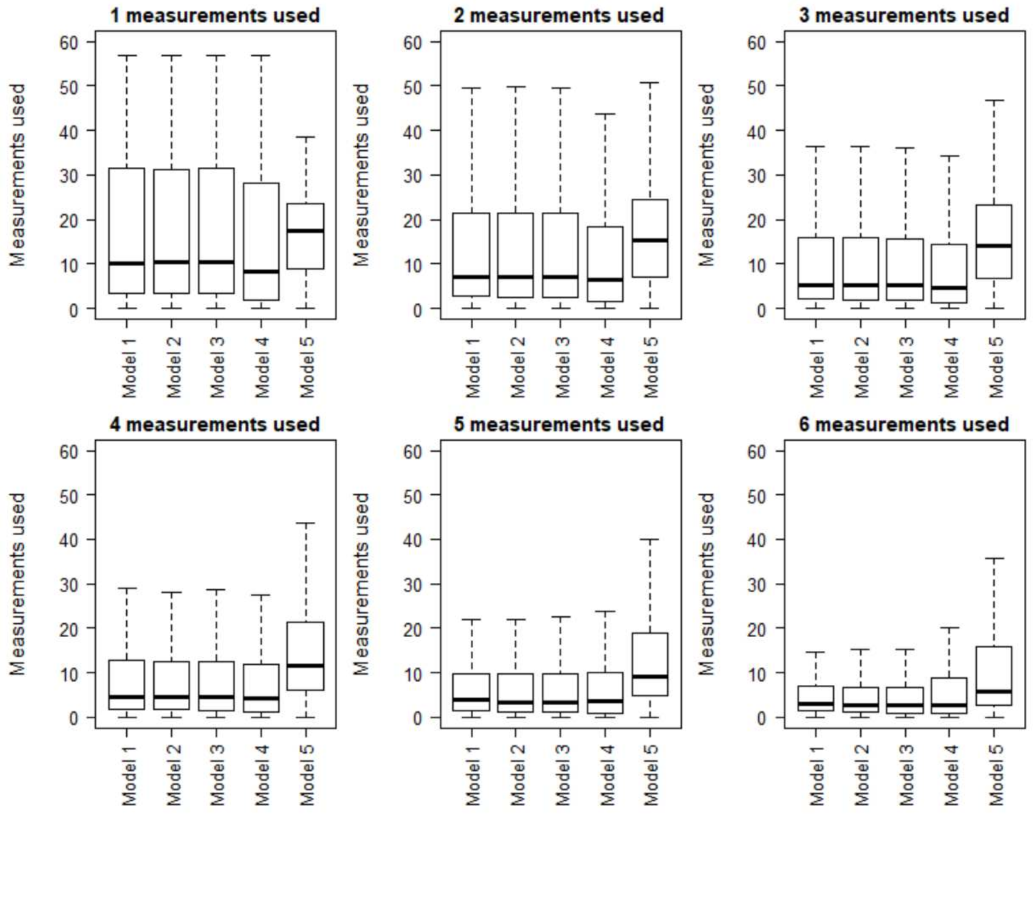

Supplementary Figure 3: the same predictions as shown in Figure 2, but with two additional patients. The data of the same patients can be downloaded in the online app to visualized predictions at all time points:
https://emcbiostatistics.shinyapps.io/DynamicPredictionARATapp/

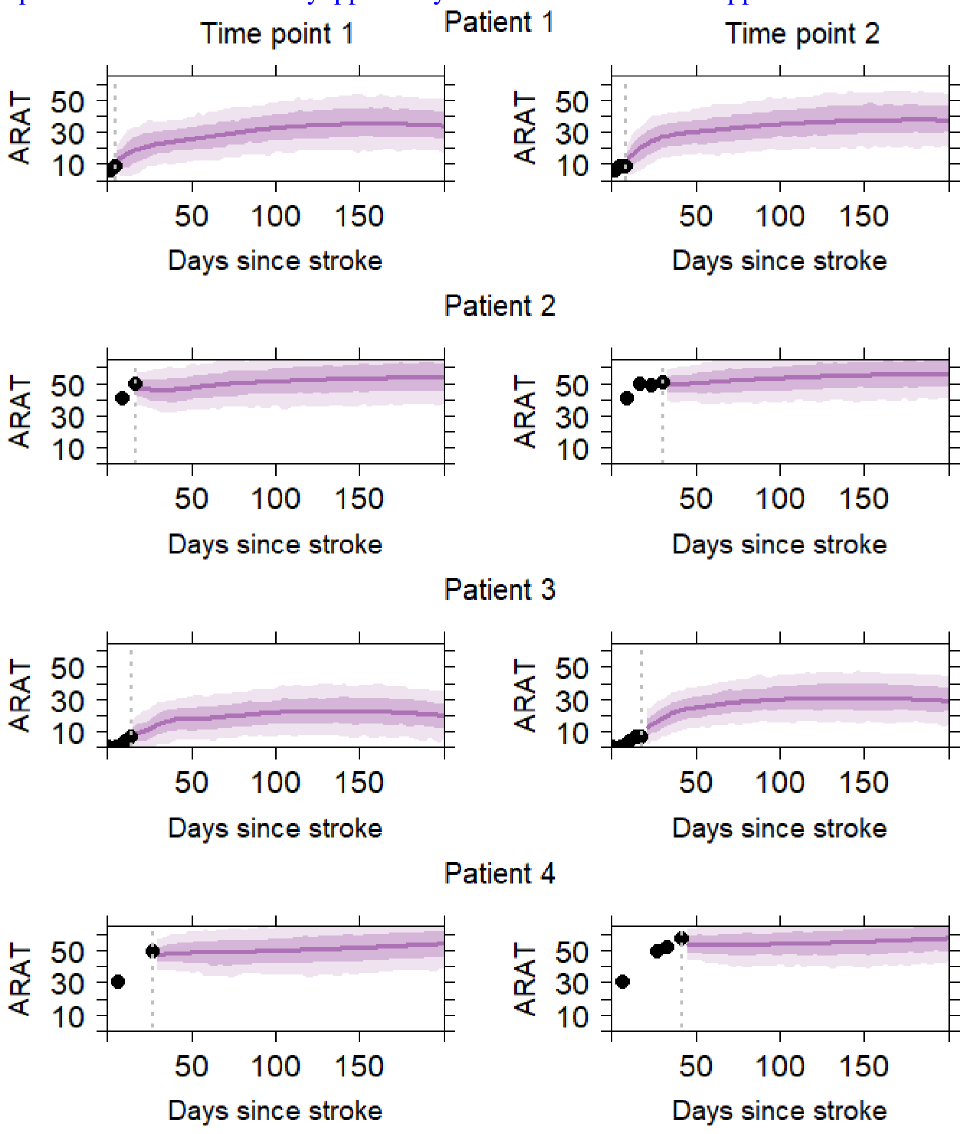

R code:

```
#########################
# Packages to install and load #
#########################

install.packages("nlme")
install.packages("splines")
install.packages("lattice")
library(nlme)
library(splines)
library(lattice)

########################################
# Functions needed for the dynamic predictions #
########################################

# The following functions were taken from the JMbayes package.
# Small changes have been made.

IndvPred_lme <- function(lmeObject, newdata, timeVar, times = NULL, M = 200L,
                         interval = c("confidence", "prediction"),
                         all_times = FALSE,
                         level = 0.95, return_data = FALSE, seed = 1L) {
  if (!inherits(lmeObject, "lme") && !inherits(lmeObject, "lmeComponents"))
    stop("Use only with 'lme' or 'lmeComponents' objects.\n")
  interval <- match.arg(interval)
  if (inherits(lmeObject, "lme")) {
    data <- lmeObject$data
    formYx <- formula(lmeObject)
    mfX <- model.frame(terms(formYx), data = data)
    TermsX <- attr(mfX, "terms")
    formYz <- formula(lmeObject$modelStruct$reStruct[[1]])
    mfZ <- model.frame(terms(formYz), data = data)
    TermsZ <- attr(mfZ, "terms")
    idVar <- names(lmeObject$modelStruct$reStruct)
    betas <- fixef(lmeObject)
    sigma <- lmeObject$sigma
    D <- lapply(pdMatrix(lmeObject$modelStruct$reStruct), "*", sigma^2)[[1]]
    V <- vcov(lmeObject)
    times_orig <- data[[timeVar]]
    times_orig <- times_orig[!is.na(times_orig)]
  } else {
    formYx <- lmeObject$formYx
    TermsX <- lmeObject$TermsX
    formYz <- lmeObject$formYz
    TermsZ <- lmeObject$TermsZ
    idVar <- lmeObject$idVar
    betas <- lmeObject$betas
    sigma <- lmeObject$sigma
    D <- lmeObject$D
    V <- lmeObject$V
    times_orig <- lmeObject$times_orig
  }
```

```
# drop missing values from newdata
all_vars <- unique(c(all.vars(TermsX), all.vars(TermsZ)))
newdata_nomiss <- newdata[complete.cases(newdata[all_vars]), ]
mfX_new <- model.frame(TermsX, data = newdata_nomiss)
X_new <- model.matrix(formYx, mfX_new)
mfZ_new <- model.frame(TermsZ, data = newdata_nomiss)
Z_new <- model.matrix(formYz, mfZ_new)
na_ind <- attr(mfX_new, "na.action")
y_new <- model.response(mfX_new, "numeric")
if (length(idVar) > 1)
  stop("the current version of the function only works with a single grouping variable.\n")
if (is.null(newdata[[idVar]]))
  stop("subject id variable not in newdata.")
id_nomiss <- match(newdata_nomiss[[idVar]], unique(newdata_nomiss[[idVar]]))
n <- length(unique(id_nomiss))

modes <- matrix(0.0, n, ncol(Z_new))
post_vars <- DZtVinv <- vector("list", n)
for (i in seq_len(n)) {
  id_i <- id_nomiss == i
  X_new_id <- X_new[id_i, , drop = FALSE]
  Z_new_id <- Z_new[id_i, , drop = FALSE]
  Vi_inv <- solve(Z_new_id %*% tcrossprod(D, Z_new_id) + sigma^2 * diag(sum(id_i)))
  DZtVinv[[i]] <- tcrossprod(D, Z_new_id) %*% Vi_inv
  modes[i, ] <- c(DZtVinv[[i]] %*% (y_new[id_i] - X_new_id %*% betas))
  t1 <- DZtVinv[[i]] %*% Z_new_id %*% D
  t2 <- DZtVinv[[i]] %*% X_new_id %*% V %*%
    crossprod(X_new_id, Vi_inv) %*% Z_new_id %*% D
  post_vars[[i]] <- D - t1 + t2
}
fitted_y <- c(X_new %*% betas) + rowSums(Z_new * modes[id_nomiss, , drop = FALSE])

if (is.null(times) || !is.numeric(times)) {
  times <- seq(min(times_orig), max(times_orig), length.out = 100)
}
id <- match(newdata[[idVar]], unique(newdata[[idVar]]))
last_time <- tapply(newdata[[timeVar]], id, max)
times_to_pred <- lapply(last_time, function (t)
  if (all_times) times else times[times > t])
id_pred <- rep(seq_len(n), sapply(times_to_pred, length))
#newdata_pred <- newdata_pred[id_pred, ]
newdata_pred <- right_rows(newdata, newdata[[timeVar]], id, times_to_pred)
newdata_pred[[timeVar]] <- unlist(times_to_pred)
mfX_new_pred <- model.frame(TermsX, data = newdata_pred, na.action = NULL)
X_new_pred <- model.matrix(formYx, mfX_new_pred)
mfZ_new_pred <- model.frame(TermsZ, data = newdata_pred, na.action = NULL)
Z_new_pred <- model.matrix(formYz, mfZ_new_pred)
predicted_y <- c(X_new_pred %*% betas) +
  rowSums(Z_new_pred * modes[id_pred, , drop = FALSE])
set.seed(seed)
betas_M <- MASS::mvrnorm(M, betas, V)
modes_fun <- function (betas) {
  t(mapply("%*%", DZtVinv, split(y_new - X_new %*% betas, id_nomiss)))
}
modes_M <- lapply(split(betas_M, row(betas_M)), modes_fun)
```

```
  matrix_row <- function (m, i) m[i, , drop = FALSE]
  modes_M <- lapply(seq_len(n), function (i) t(sapply(modes_M, matrix_row, i = i)))
  b_M <- modes_M
  for (i in seq_len(n)) {
    b_M[[i]] <- t(apply(modes_M[[i]], 1, MASS::mvrnorm, n = 1, Sigma = post_vars[[i]]))
  }
  n_pred <- length(predicted_y)
  sampled_y <- matrix(0.0, n_pred, M)
  for (m in seq_len(M)) {
    betas_m <- betas_M[m, ]
    b_m <- t(sapply(b_M, function (x) x[m, ]))
    mean_m <- c(X_new_pred %*% betas_m) +
      rowSums(Z_new_pred * b_m[id_pred, , drop = FALSE])
    sampled_y[, m] <- if (interval == "confidence") mean_m
    else rnorm(n_pred, mean_m, lmeObject$sigma)
  }
  low <- apply(sampled_y, 1, quantile, probs = (1 - level) / 2)
  upp <- apply(sampled_y, 1, quantile, probs = 1 - (1 - level) / 2)
  rm(list = ".Random.seed", envir = globalenv())
  if (!return_data) {
    list(times_to_pred = times_to_pred, predicted_y = predicted_y,
         low = low, upp = upp)
  } else {
    out_data <- rbind(newdata, newdata_pred)
    out_data$pred <- c(fitted_y, predicted_y)
    out_data$low <- c(rep(NA, length(fitted_y)), low)
    out_data$upp <- c(rep(NA, length(fitted_y)), upp)
    out_data[order(out_data[[idVar]], out_data[[timeVar]]), ]
  }
}

right_rows <- function(data, times, ids, Q_points) {
  fids <- factor(ids, levels = unique(ids))
  if (!is.list(Q_points))
    Q_points <- split(Q_points, row(Q_points))
  ind <- mapply(findInterval, Q_points, split(times, fids))
  ind[ind < 1] <- 1
  rownams_id <- split(row.names(data), fids)
  ind <- mapply(`[`, rownams_id, split(ind, col(ind)))
  data[c(ind), ]
}

############################
# Function for the dynamic plots #
############################

DynPlots <- function(model.output = model.output, newdata, timeVar,
                     main_title = "Dynamic predictions"){

  # Load individual prediction ------------------------------------
  data <- model.output$data
  formYx <- formula(model.output)
  yOutcome <- formYx[[2]]

  IndvPrediction95 <- IndvPred_lme(lmeObject = model.output, newdata, timeVar,
```

```
times = NULL, M = 500, interval = "prediction", return_data = TRUE)

  IndvPrediction68 <- IndvPred_lme(lmeObject = model.output, newdata, timeVar,
times = NULL, M = 500, interval = "prediction", return_data = TRUE, level = 0.68)

  pred95 <- IndvPrediction95[which(!is.na(IndvPrediction95$low)),]
  pred68 <- IndvPrediction68[which(!is.na(IndvPrediction68$low)),]

  nopred <- IndvPrediction95[which(is.na(IndvPrediction95$low)),]

  timeVariable <- pred95[[timeVar]]

  # Generating plot ----------------------------------------------------
  xyplot(pred ~ timeVariable , main = main_title, data = pred95,
       type = "l", col = rgb(0.6769,0.4447,0.7114, alpha = 1), lty = c(1, 2, 2), lwd = 3,
       ylim = c(0,65), xlim = c(0,200), ylab = list(yOutcome, cex = 1.5), xlab = list(timeVar, cex
= 1.5),
       scales = list(x = list(cex = 1.3) , y = list(cex = 1.3)),
       panel = function(x, y,  ...) {
         panel.xyplot(x, y, ...)
         panel.polygon(c(pred95[,"Days"], rev(pred95[,"Days"])),
                       c(pred95[,"upp"], rev(pred95[,"low"])),
                       border = NA,
                       col = rgb(0.6769,0.4447,0.7114, alpha = 0.2))
         panel.polygon(c(pred68[,"Days"], rev(pred68[,"Days"])),
                       c(pred68[,"upp"], rev(pred68[,"low"])),
                       border = NA,
                       col =rgb(0.6769,0.4447,0.7114, alpha = 0.4))
         panel.points(x = nopred[[timeVar]], y = nopred[[yOutcome]], cex = 1.2, pch = 16, col =
"black");
         panel.lines(x = rep(tail(nopred[[timeVar]], n = 1), 200), y = seq(-100, 100, length =
200), col = "grey", lty = 3, lwd = 2)
       })
  }

###########################
# Run the mixed-effects model #
###########################

# ARAT: Action Research Arm test
# SA: shoulder abduction, defined as:
#      - no random movement
#      - random activity palpable, no movement visible
#      - random movement see able but not seeable in total movement range
#      - random movement across total movement range, not possible against resistance
#      - random movement agains resistance , but weaker than 'healthy' side
#      - normal strength in comparison with 'healthy' side
# FE: finger extension, defined as:
#   - none
#   - partial
#   - full
# Days: The day were the measurements of the ARAT, SA and FE were taken
# Number: is the identification number
# data: include all the variables mentioned above
```

```
model_SA_FE <- lme(ARAT ~  SA * ns(Days, knots = c(6, 11, 19, 34, 50, 91)) +
                                  FE * ns(Days, knots = c(6, 11, 19, 34, 50, 91)),
     data = data,
                       random = list(Number =
                                  pdDiag(form = ~  ns(Days, knots = c(6, 11, 19, 34, 50,
91)))),
                       na.action = na.exclude,
                       control = lmeControl(maxIter = 1e8, msMaxIter = 1e8))

##############################
# Obtain the dynamic predictions #
##############################
# Define a new patient or select one from the data set
nd <- data[data$Number == 1, ]
# Obtain the plot
DynPlots(model.output = model_SA_FE, newdata = nd,
          timeVar = "Days",
          main_title = "Patient")
```
